# A Different View for an Old Disease: NEDDylation and Other Ubiquitin-Like Post-Translational Modifications in Chronic Lymphocytic Leukemia

**DOI:** 10.3389/fonc.2021.729550

**Published:** 2021-09-23

**Authors:** Víctor Arenas, Jose Luis Castaño, Juan José Domínguez-García, Lucrecia Yáñez, Carlos Pipaón

**Affiliations:** Laboratorio de Hematología Molecular, Servicio de Hematología, Hospital Universitario Marqués de Valdecilla-Instituto de Investigación Marqués de Valdecilla (IDIVAL), Santander, Spain

**Keywords:** chronic lymphocytic leukemia, post-translational modifications, ubiquitin, NEDD8, MLN4924-pevonedistat

## Abstract

Despite the enormous amount of molecular data obtained over the years, the molecular etiology of chronic lymphocytic leukemia (CLL) is still largely unknown. All that information has enabled the development of new therapeutic approaches that have improved life expectancy of the patients but are still not curative. We must increase our knowledge of the molecular alterations responsible for the characteristics common to all CLL patients. One of such characteristics is the poor correlation between mRNA and protein expression, that suggests a role of post-translational mechanisms in CLL physiopathology. Drugs targeting these processes have indeed demonstrated an effect either alone or in combination with other aimed at specific pathways. A recent article unveiled an increment in ubiquitin-like modifications in CLL, with many protein members of relevant pathways affected. Interestingly, the inhibition of the NEDD8-activating protein NAE reverted a substantial number of those modifications. The present review gets the scarce data published about the role of NEDDylation in CLL together and establishes connections to what is known from other neoplasias, thus providing a new perspective to the underlying mechanisms in CLL.

## Chronic Lymphocytic Leukemia

Chronic lymphocytic leukemia (CLL) is the most frequent lymphoid malignancy in the Western world and appears typically in the elderly. The diagnosis of this disease requires the presence of ≥5 x10^9^/L monoclonal mature CD19+ CD5+ B cells with a characteristic phenotype that usually infiltrates the blood, bone marrow and lymphoid tissues (1). CLL has a very heterogeneous clinical course: some patients show a stable course without the requirement for therapy, while others show a more aggressive disease requiring early treatment and, in some cases, turning into an aggressive lymphoma (Richter transformation) (1). In the last 30 years, many aspects on its biology have been unraveled. The discovery of the dysregulation of several survival pathways or the importance of BCR signaling in the maintenance of the tumoral cells (2), has uncovered new therapeutic targets. Nevertheless, our knowledge about the causes of this chronic disease is still quite poor.

## Physiopathology of CLL

The physiopathology of CLL is a complex process that can be explained in part by a sequence of events that lead to the transformation of a normal B CD5+ cell into a neoplastic cell. The starting point of the disease is not well known. The fact that CLL develops in the adult population suggests that aging-related factors, unknown at present, play an essential role in the pathogenesis of the disease. In approximately 10% of the patients, a genetic predisposition may explain the development of CLL (3). Other initiation factors such as a dysfunction on the immune system related to some infections or the exposition to certain toxics have been speculated, but to date none of them has been reliably verified.

In cancer, the neoplastic transformation usually starts with an alteration of the chromosomal material and in approximately 80% of patients with CLL at least one of four common chromosomal abnormalities (Chromosome 13q deletion, chromosome 12 trisomy, chromosome 11q deletion and chromosome 17p deletion) can be detected by interphase fluorescence *in situ* hybridization (FISH) at the moment of diagnosis or in their evolution. A model of sequential chromosomal abnormalities and genetic mutations has been proposed, defining early, intermediate and late drivers that allow CLL cells to develop and evolve. Chromosome 13q deletion, chromosome 12 trisomy and mutations in MYD88 or NOTCH1 are considered early drivers of the disease while the acquisition of chromosome 11q or chromosome 17p deletions as well as mutations in genes frequently mutated in cancer (TP53, ATM, SF3B1 or NRAS) appear during CLL progression (4).

The microenvironment has also been related to the proliferation, survival, and drug resistance in B cell neoplasms like multiple myeloma, mantle cell lymphoma and specifically CLL. Mesenchymal cells, nurse-like and lymphoma-associated macrophagic cells, natural killer and other T cells, interact with CLL neoplastic cells engaging some receptors like BCMA, TACI, BAFF-R or VLA-4, thus activating anti-apoptotic pathways and promoting the secretion of cytokines, chemokines, growth factors and adhesion molecules allowing the extended survival of the malignant B cell (5).

## Genomic Studies in CLL

Over the last years, a great effort has been put in trying to identify the genomic causes of CLL. As a result of these studies, several genes have been identified as involved in the pathogenesis of the disease. Among those are TP53, NOTCH1, BIRC3, SF3B1 or ATM (2, 6, 7). Nevertheless, the mutations affecting them do not exceed a 15% of penetration, so there are still many issues to be solved. Taking into account the somatic mutations, the cytogenetic alterations and the mutations in non-coding regions, they conclude CLL relates to alterations in eight signaling pathways: B cell receptor, cell cycle regulation, apoptosis, response to DNA damage, chromatin remodeling, RNA metabolism and Notch and NF-kB pathways (7).

## Current Therapies

For many years, chemotherapy was the treatment of choice for patients with CLL. However, the improvement in the knowledge of the molecular processes involved in CLL pathogenesis has marked current therapeutic strategies. The discovery of the importance of B-cell antigen receptor (BCR) signaling in CLL cells completely changed the landscape in the treatment of this disease. BCR engagement leads to a massive recruitment of kinases to the plasma membrane that transduce the signal to many effector pathways and transcription factors essential for B lymphocyte survival and proliferation. To date, drugs against phosphoinositide 3-kinase (PI3K) (idelalisib, duvelisib, copanlisib, umbralisib) and Bruton´s Tyrosine Kinase (BTK) (ibrutinib, acalabrutinib, zanubrutinib, pirtobrutinib) have been studied, and some approved, for the therapy of patients with CLL with excellent results, not only in progression free survival but also in overall survival, in comparison to standard chemoimmunotherapy (8).

The involvement of Bcl-2 in the prolonged survival of B-CLL cells is long known (9). However, the toxicity related to the first in class Bcl-2 inhibitor, navitoclax, prevented this drug from being used in the past. Recently, a new Bcl-2 specific inhibitor, venetoclax, has demonstrated a huge activity against B-CLL cell survival with an excellent toxicity profile and nowadays is broadly used to achieve a deep response that allow to stop the treatment (10).

## NEDDylation

**Figure 1:**
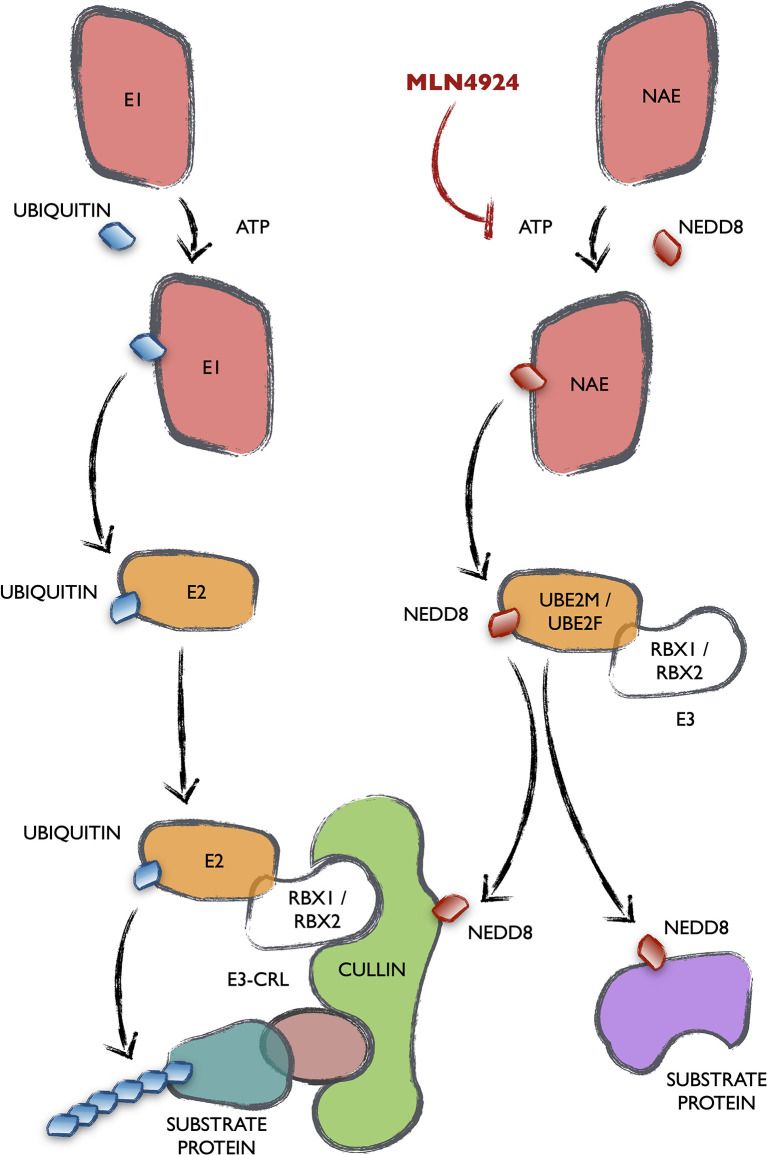
Mechanism and connection of the ubiquitination and the NEDDylation cascades. All UBLs are conjugated to their target proteins following a completely homologous cascade in three steps catalyzed by enzymes generally termed as E1, E2 and E3. The names of the enzymes specific of the NEDDylation cascade are stated (NAE, UBE2M/UBE2F as well as its best characterized E3s RBX1/RBX2). The main NEDDylation target proteins are Cullins, that form part of one of the biggest groups of E3 ubiquitin ligases, the Cullin-RING ligases (CRLs).

NEDDylation is a post-translational modification consisting in the covalent conjugation of a NEDD8 peptide with target proteins at lysine residues. NEDD8 is one of the ubiquitin-like peptides (UBLs), with 80% homology to ubiquitin and an activation and conjugation mechanism absolutely analogous (**Figure 1**). This occurs through the sequential action of three enzymes generally termed as E1, E2 and E3. NAE is the specific E1 that activates NEDD8 and is the target of an investigational inhibitor named MLN4924-Pevonedistat. Activated NEDD8 is then transferred to the E2s UBE2M or UBE2F. Different target-specific E3 proteins adapt the system for the modification of many different proteins, thus affecting a wide array of cellular processes. The majority of NEDD8 E3 ligases contain a RING domain, including RBX1, RBX2, c-CBL, FBXO11, IAP, MDM2, TRIM40, RNF111 and TFB3. Noteworthy, the existence of isopeptidases that remove NEDD8 from target proteins underlines the regulatory essence of this process. Among them, only NEDP1 and the COP9 signalosome are NEDD8-specific (11). There is a close connection between the ubiquitination and NEDDylation processes, since the major group of E3 ubiquitin ligases, the Cullin-RING ligases (CRLs), must have their Cullin component NEDDylated to be functional (12, 13). Although Cullins are the main target of NEDDylation, many cellular processes have been reported to be regulated by the conjugation of NEDD8 to some of their effector proteins (14).

## Role of NEDDylation in CLL

NEDDylation is a post-translational modification that affects many critical aspects of cancer (15, 16). Due to this, MLN4924-pevonedistat is being tested in clinical trials for the treatment of different types of tumors (https://clinicaltrials.gov/ct2/results?term=MLN4924&Search=Search). Our knowledge of the role of NEDDylation in the physiopathology of CLL comes from the reported action of MLN4924 in ex-vivo experiments, as well as in other types of cancer. These studies have identified several target pathways in CLL.

As already mentioned, B-CLL cells are highly dependent on cell to cell interactions in bone marrow and lymphoid organs. In these niches, nuclear factor-κB (NF-κB) signaling gets activated in B-CLL cells, ensuring apoptosis evasion (17). It has been shown that MLN4924 successfully truncates NF-κB pathway activity, B-CLL cell survival and chemoresistance in an *in vitro* co-culture model that mimics the lymph node microenvironment. MLN4924 extends the half-life of the inhibitor of NF-κB (IκB), a negative modulator of the pathway, by preventing its polyubiquitination and degradation (18, 19).

Earlier findings suggested that CLL cells possess DNA damage repair ability that is highly variable between individual samples (20, 21). Contrarily, others reported a defect in the repair of UVC-induced lesions in CLL cells (22), suggesting an affection of specific DNA repair pathways in CLL. Treatment with MLN4924 leads to an accumulation of the licensing factor Cdt1 and sensitization to alkylating agents in B-CLL cells (23). Overexpression of Cdt1 has been shown to induce DNA re-replication followed by head-to-tail collision of replication forks (24).

## Distribution of the Ubiquitin-Like Modifications in CLL

As already mentioned, several groups have shown a cytostatic effect of MLN4924 over B-CLL cells ex vivo, potentiating the action of alkylating agents and drugs commonly used in the treatment of this neoplasia, like ibrutinib and idelalisib (18, 23, 25). Nevertheless, the extension and relevance of NEDDylation in these cells is unknown. To shed some light on this respect, we recently used the Ubiscan platform, that utilizes an antibody against the di-glycine remnants left over the modified lysines when proteins are digested with trypsin (26). The peptides thus enriched were then identified by mass spectroscopy. However, the homology between ubiquitin, NEDD8 and ISG15 in their N terminus makes it impossible to distinguish each of these types of modifications using this methodology. Akimov et al. developed a ubiquitination-specific antibody for the profiling of whole cell samples treated with the endopeptidase Lys-C and reported a ubiquitination profile of Jurkat and Hep2 cell lines (27). Although some reports have tried to establish a consensus NEDDylation target sequence that would allow to discriminate exclusive NEDDylation sites (28), this is still largely unknown, so we could not use Akimov’s work as a reference to exclude ubiquitination sites from our screening. On the other hand, two different groups have recently developed strategies to identify proteome-wide NEDD8 modification sites in HEK293T and HCT116 human cell lines (29, 30). Unfortunately, the genetic alteration methods needed make them not suitable for the profiling of primary samples from patients.

In an attempt to circumvent those drawbacks, we analyzed matched samples treated or not with MLN4924, to distinguish NEDDylation-sensitive sites in CLL. The fact that cullins and other previously identified NEDDylated sites reverted their modification level with the treatment, validated the approach. Even so, as the NEDDylation of Cullins regulates CRL function, the modifications found could still be NEDDylation-sensitive ubiquitinations. In concordance with those works reporting a somehow correcting effect of MLN4924 over B-CLL cells, our screening identified a preponderance of peptides with an increased rather than decreased MLN4924-sensitive modification, supporting the idea that NEDDylation is elevated in chronic lymphocytic leukemia. We identified 353 lysines with a significant increment in their di-Gly remnant modification (2.5 fold threshold) that was reverted with MLN4924, thus making them candidates to be direct NEDDylations. We compared those NEDDylation candidate sites to the bona fide NEDDylation sites obtained in the screenings of Vogl et al. and Lobato-Gil et al. (29, 30) in search for some kind of confirmation. 23 and 14 sites coincide with each of the screenings, respectively. Only 5 lysines were common to all three screenings: NEDD8 K22, PPIA K125, PARP1 K97, UBA3 K409 and HMGB1 K114. When comparing the proteins target of the modifications, we found 20 coincidences between the three studies: 34 and 62 common target proteins between our study and each of those screenings, respectively. These data, emphasizes the cell-type specific profiles of NEDDylation but does not exclude other nature for the UBL modifications found in CLL. Interestingly, 5 of the NEDD8 lysines found in B-CLL cells showed a discreet increment in their modification, that was clearly reverted upon incubation with MLN4924 (31), indicating the generation of poly-NEDD8 chains in CLL. Only lysine 11 and, to a lesser extent, lysine 5 of SUMO2, but no ubiquitin residue, were represented in our screening, indicating putative alterations in the NEDD8-SUMO2 but not in the NEDD8-ubiquitin chains in CLL. These data are in concordance with those pointing at K11 of SUMO2 as a key residue in the generation of NEDD8-SUMO2 hybrid chains (30).

Deletion 2q37 is the genomic alteration that confers the highest augmented overall survival-hazard ratio in CLL (7). Interestingly, this region harbors the loci for three members of the COP9 signalosome complex: COPS8, COPS7B and COPS9. Additionally, COPS5P1 locus maps in 6q16.1, a region which deletion is also related to an increase in survival hazard (7). Theoretically, deletion of these members of the COP9 signalosome, involved in the deNEDDylation of Cullins, may result in the overall increase in protein post-translational modifications that we observed in the scan, supporting a key role of NEDDylation in the cellular phenotype of CLL. In addition, although the mononuclear fraction of patients and controls is not completely comparable, the data showed an over-expression of the genes coding for the E1 and E2 proteins specific of the NEDD8 activation pathway (NAE1, UBE2F and UBE2M) in CLL patients (31).

**Figure 2:**
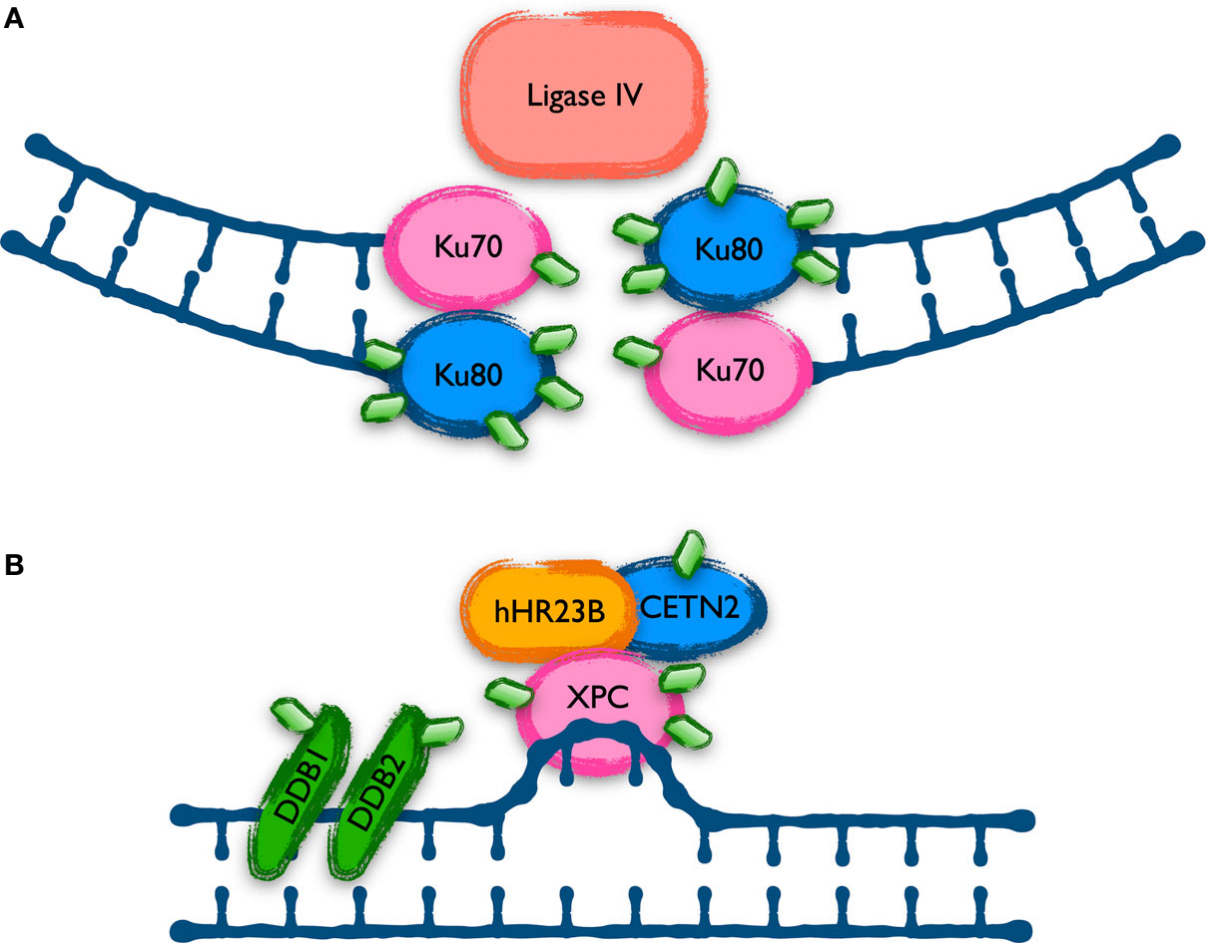
Proteins involved in DNA repair mechanisms with UBL-PTM affected in CLL. Cells repair different types of DNA damage using specific mechanisms. Proteins involved in two of these mechanisms show an altered UBL modification level in CLL. **(A)** The Non-Homologous End Joining (NHEJ) is the main mechanism to repair double strand breaks in human cells but is prone to introduce mutations. Ku70 and Ku80 play a key role in the re-joining of the broken ends, and they are aberrantly modified in CLL, as shown with green diamonds in the scheme. **(B)** The Nucleotide Excision Repair (NER) system is in charge of repairing lesions generating mismatches in the DNA, like those induced by UV irradiation. Previous data indicated a defect in NER in CLL and the recent data shows alterations in the level of UBL modification of some of the proteins involved, as represented in the figure with green diamonds.

The analysis of the UBL-PTM data by intracellular pathways fits very well with the mutational studies and the cellular manifestations of the disease. However, a detailed characterization of the nature and effects of the modifications found in the harboring proteins is needed to determine their contribution to the CLL phenotype. For some of those pathways there was reported evidence of the role of NEDDylation in their regulation. This is the case of the non-homologous end joining DNA repair mechanism of double strand breaks (DSB), involved in some resistances of CLL cells (32), where NEDDylation is needed for the release of Ku70 and Ku80 from DNA (33) (**Figure 2A**). These two proteins are profusely over-modified in CLL and MLN4924 reverts in part those modifications. Ku70 and Ku80 stabilize the DNA ends generated when double strand breaks occur and recruit ligase IV (XRCC4) to rejoin them. This mechanism is error prone, and it might be behind the cytogenetic alterations selected in CLL. By contrast, proteins involved in the repair of DSB by homologous recombination are scarcely represented in the screening of UBL-PTM in CLL, being lysine 765 of FANCB an exception. FANCB participates in the E3 complex that monoubiquitinates FANCD2 and FANCI as part of the detection and signaling of DSB, but none of those proteins showed variations in their modifications, maybe suggesting a role of FANCB in CLL aside of the FANCcore complex. In that direction, overexpression of FANCA have been found as a bad prognosis factor in CLL, since it collaborates with MDM2 in the destabilization of p53 (34). Moreover, FANCA is necessary in the NEDDylation of several CLL markers like CXCR5, beta-2-microglobulin and CD44 (35). Apart from the repair of DSB, several members of the nucleotide excision repair (NER) machinery, specialized in the repair of mismatched DNA bases like those produced by UV radiation, were found to be highly modified with MLN4924-sensitive UBLs in CLL (**Figure 2B**). Distortions in the DNA helix are directly recognized by XPC, complexed with hRAD23B and centrin 2 (CETN2). Lesions that do not destabilize DNA duplexes are first recognized by DDB2 (XPE) in complex with DDB1, creating a kink that is then recognized by XPC. DDB1 and DDB2 are part of the CUL4-ROC1 ubiquitin ligase complex that ubiquitinates DDB2, XPC and several histones upon DNA damage. The XPC-hRAND23b-CETN2 complex melts the DNA around the lesion and attracts the rest of the NER repair proteins. The UBL modification of these proteins involved in the recognition of DNA lesions might be related to the hypersensitivity of B-CLL cells to UVC radiation (22, 36). Whether these modifications collaborate in the generation of mutations or cytogenetic aberrations leading to the progression of CLL is still to be determined.

**Figure 3:**
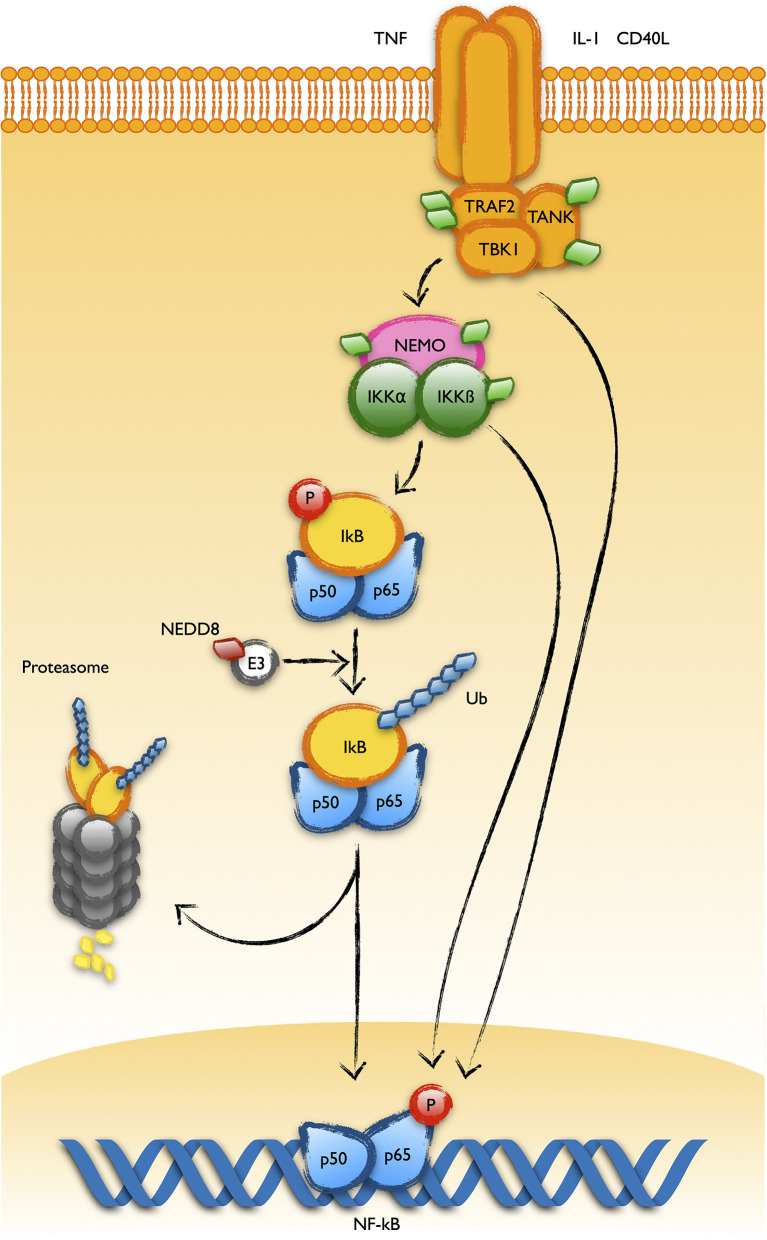
UBL-PTMs of the NF-kB pathway altered in CLL. Schematic representation of the main components of the NF-kB pathway showing the ubiquitin-like post-translational modifications found in B cells from patients of CLL. It was long known that activation of the pathway involves the polyubiquitination of the inhibitor IkBa and its degradation at the proteasome. This process is regulated by NEDDylation of the Cullin component of the E3 ubiquitin ligase targeting IkB for its polyubiquitination. Recent observations in B-CLL cells have revealed alterations in the post-translational pattern of proteins working upstream in the pathway, like TRAF2, TANK, NEMO or IKKß (represented as green diamonds in the figure).

The NF-kB pathway is a well stablished target of the action of MLN4924, supporting a key role of NEDDylation in its regulation (19, 37). The group of Doctor Danilov have found that MLN4924 thwarts the survival support provided over B-CLL cells by the stroma of lymphoid organs through its negative regulation of the NF-kB pathway (18, 38). NF-kB signaling inhibition in T cells also seems to play a role in the immunologic anergy of CLL (39). Since activation of the NF-kB pathway by the canonical way involves the degradation of the inhibitor IkB by the Skp1-Cul1-F-box (SCF^Fbw1^) complex, and given the regulation exerted by NEDDylation over this later, IkB stabilization has been assumed as the interaction level of MLN4924 over the pathway. Of note, several works have demonstrated the association of the COP9 signalosome and the IKK complexes, facilitating the coupled phosphorylation and degradation of IkB, central in the activation of the pathway (40, 41). However, the MLN4924-sensitive modifications found in the UBL-PTM profile of CLL reveals other putative points where the drug may be exerting its action. Thus, the upstream proteins TANK, TRAF2, CD40, NEMO and IKKß are aberrantly modified in B-CLL cells, suggesting other levels of regulation of this pathway by NEDDylation (**Figure 3**). Interestingly, NEDDylation of NEMO by the RING E3 ligase TRIM40 in the gastrointestinal tract inhibits NF-kB activity and TRIM40 is down regulated in gastrointestinal carcinomas (42). In addition, MLN4924 induces phosphorylation of IkB as well as p65 by itself, keeping total p65 levels unchanged (43).

**Figure 4:**
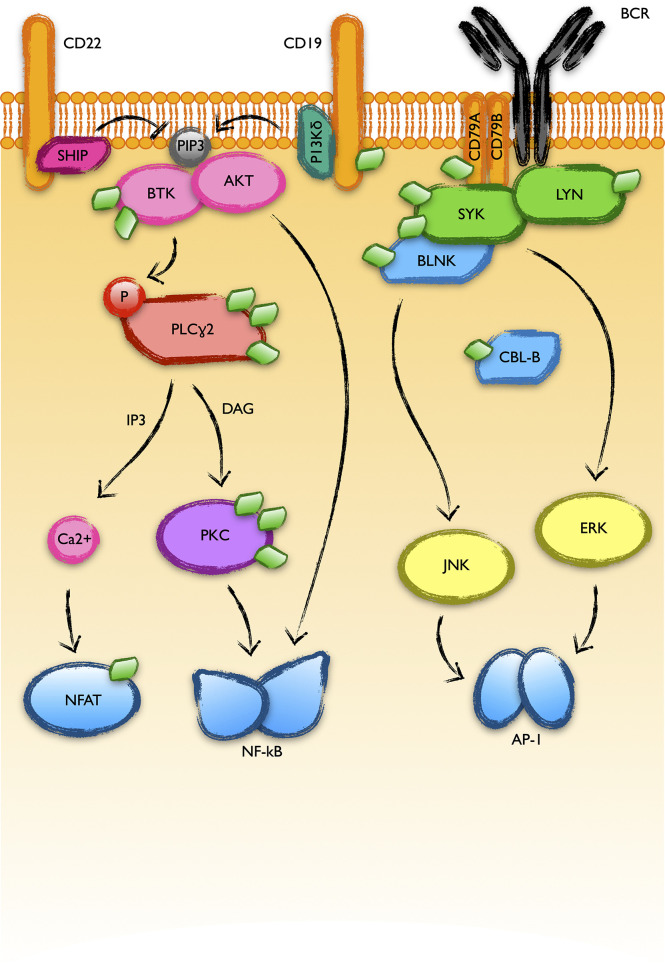
BCR-activated pathways with UBL-PTMs altered in CLL. B cell receptor (BCR) engagement triggers a massive recruitment of protein kinases to the plasma membrane where they get activated and transduce the signal to final transcription factor effectors like NFAT, NF-kB or AP-1. The good response of B-CLL cells to inhibitors of BTK or PI3K-delta supports a relevant role of these pathways in the physiopathology of CLL. The recent screening of the UBL-PTM in CLL showed alterations in the modifications of many proteins involved in these pathways (represented as green diamonds in the figure).

As previously mentioned, current therapies to fight CLL target the pathways downstream of the B cell receptor (BCR), as well as the anti-apoptotic protein Bcl-2. BCR drives the B-cell response to the presentation of antigens, triggering a bunch of survival pathways that lead to the expansion of the clone to fight infection. Due to reasons still not fully understood, BCR signaling is elevated in CLL. Upon binding to an antigen, BCR recruits a great amount of kinases to the cytoplasmic membrane, thus promoting their mutual inter-activation. This is the case of the kinases SYK and LYN. In addition, BCR signaling receives positive and negative reinforces from other membrane proteins (44): CD19 activates PI3K, which in turn phosphorylates phosphatidylinositol (4, 5)-bisphosphate that recruits BTK to the membrane where it is activated by phosphorylation (45, 46). On the other hand, membrane proteins like CD22 recruit phosphatases like SHIP, reverting the previous process (47). The recruitment of this protein complex to the membrane results in the activation of downstream survival and proliferation pathways like NF-kB or MAPK in CLL. SYK, LYN, BTK or the adaptor protein BLNK are highly modified with UBL molecules in CLL (**Figure 4**). More interestingly, these modifications coordinately increase in response to MLN4924, which discards their direct NEDD8 modification but suggests a common NEDDylation-dependent regulation. This might be an exploitable mechanism to strongly interfere with the activation of the pathway and avoid resistances based on individual mutations.

Proteins involved in many other cellular processes associated with the development or prognosis of CLL were also revealed as aberrantly modified in CLL in the screening. This is the case of proteins of the cytoskeleton, the chromatin or the RNA processing machinery, as described next.

**Figure 5:**
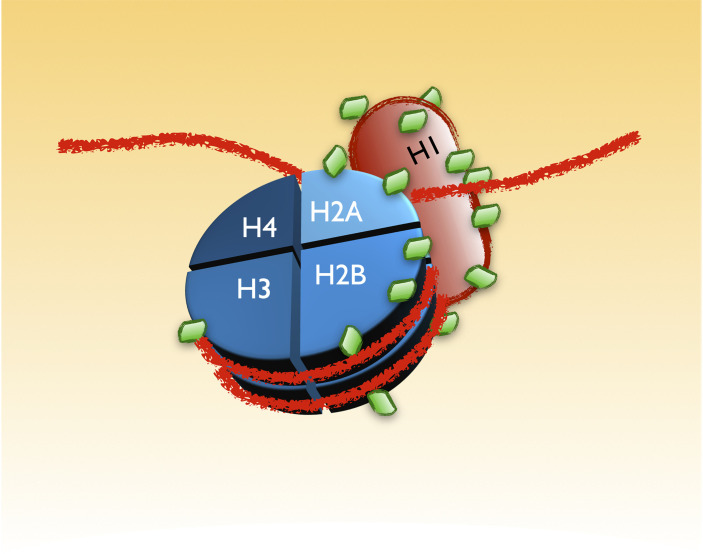
UBL-PTMs of components of the chromatin altered in CLL. B-CLL cells are characterized by a distinctive appearance of their chromatin. The recent screening revealed an extensive alteration in the pattern of UBL-PTMs of the histones that may account for the particular chromatin dynamics in these cells. The figure is a scheme of those modifications, depicted as green diamonds. As in the other figures, the amount of green diamonds on each protein represents the approximated proportion of lysines with altered modifications in CLL.

One of the most largely known alterations of B-CLL cells, still used in its diagnosis, is the condensation of the chromatin in clonal cells (48). For that reason, it was interesting to realize that the largest group of proteins with K-GG remnants sensitive to MLN4924 identified in our screening were histones and other chromatin associated proteins. As schematically seen in **Figure 5**, several histones conforming the core of the nucleosomes (H2B, H2A, H3 and specially H1) receive a higher amount of modifications in CLL (31). Interestingly, all of these modifications are reverted at different extent with 0.25µM of the NAE inhibitor, suggesting a role of NEDDylation in the characteristic chromatin dynamics of CLL.

Similarly, fragility of the B-CLL cells when smeared in microscopy preparations is another distinctive characteristic of this pathology, giving rise to broken cells named smudge cells or Gumprecht shadows. The manifestation of smudge cells in smear peripheral blood preparations of CLL patients has been associated with low vimentin levels (49, 50) and, intriguingly, with a better prognosis (51). Vimentin and other cytoskeleton proteins showed an increase in post-translational modifications in CLL and treatment with MLN4924 reverted those modifications along with a reduction in the amount of smudge cells (31). Although the study of a larger amount of samples is needed to get solid conclusions, these results suggest a negative effect of NEDDylation over vimentin and other components of the cytoskeleton, posing a role of post-translational modifications in a characteristic cellular manifestation of CLL.

**Figure 6:**
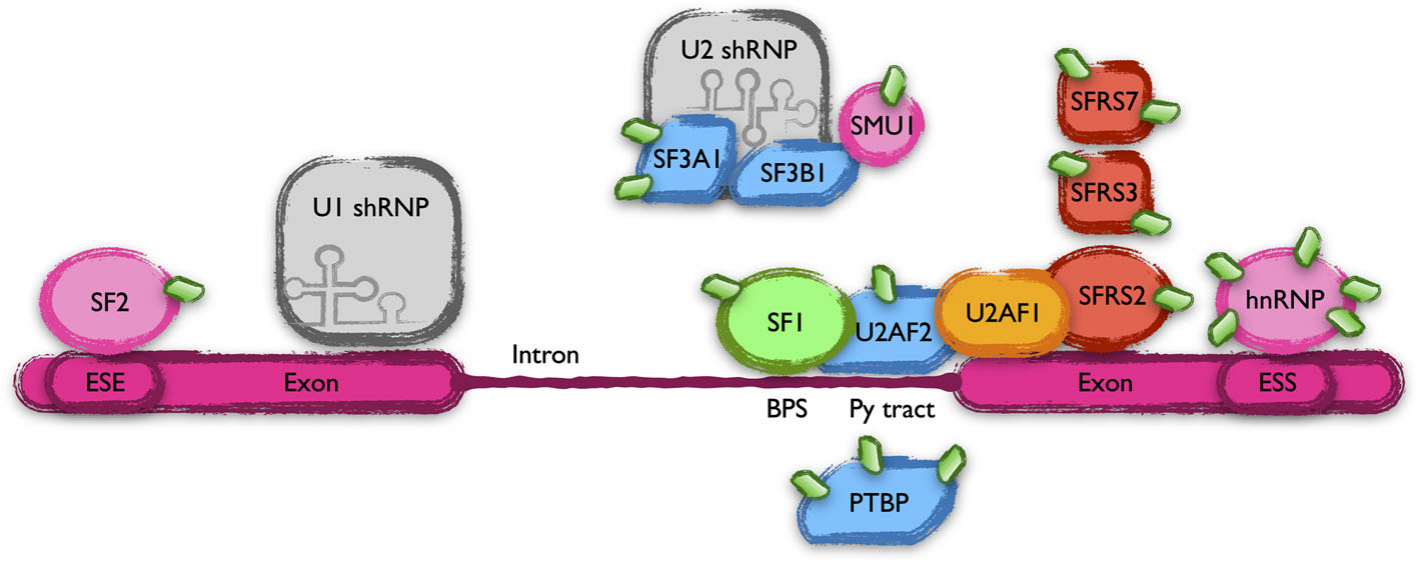
Scheme of the members of the spliceosome that harbor UBL-PTMs altered in CLL. Schematic representation of part of the spliceosome complex, focusing on the proteins with altered UBL-PTM in CLL. Introns are marked in nascent mRNAs by cis elements (BPS, Py tract, ESE, ESS, *etc*, see text) bound by an array of proteins that collaborate in their elimination.

Mutations in the splicing factor SF3B1 have been found in around 15% of CLL patients, suggesting an alteration of RNA splicing (52). Our screening found alterations in the modification state of many proteins involved in this mechanism. Eukaryotic genes include non-coding sequences called introns that are removed by a complex array of proteins known as the spliceosome. Introns are marked by sequence elements in the mRNA. These cis elements include conserved sequences at the 5’ and 3’ ends of exons as well as two other elements called the branch point sequence (BPS or BS) and the polypyrimidine (PY) tract. In addition, pre-mRNAs include exonic and intronic splicing enhancers (ESEs or ISEs) and silencers (ESSs or ISSs) that modulate constitutive and alternative splicing by binding regulatory proteins that either stimulate or repress the splicing at an adjacent site (53). Many proteins binding to these cis elements, responsible for the initial steps of spliceosome assembly, received an elevated and MLN4924-sensitive level of UBL post-translational modifications in CLL, indicating a putative NEDDylation. Among them are the BPS binding protein SF1, the U2 small nuclear ribonucleoprotein (snRNP)-associated protein SF3A1, several members of the serine/arginine (SR)-rich protein family (SFRS2, SFRS3 and SRFS7) or the ESE-binding SF2 factor (**Figure 6**). In addition, the UBL modifications of a bulky list of proteins that include PY tract binding proteins, ESE or ESS binding factors, RNA helicases or small nuclear ribonucleoproteins, among others, are altered in CLL with different sensitivities to NAE inhibition. NEDDylation of SRSF3 at lysines 11 and 85 has already been related to processes like the progression of liver disease and the assembly of stress granules in response to arsenite-induced oxidative stress, respectively (54). On the other hand, SUMOylation plays a well-known role in the regulation of the RNA processing machinery (55). Together with the presence of alterations in the modification of SUMO in lysine 11 (see above), our data may indicate a cross-talk between these two post-translational modifications in the regulation of splicing in CLL.

Constitutive activation of NOTCH is considered one of the main drivers of CLL, since it has been found in at least, but not only, the 4-13% of CLL cases carrying NOTCH1 mutations (2, 56, 57). Somehow surprisingly, no alteration in the modifications of proteins directly involved in this pathway were found in CLL. However, some modulators of this pathway are represented in our study like MIB1 (highly modified in two adjacent lysines in CLL according to our screening) that positively regulates NOTCH signaling by ubiquitinating and facilitating the endocytosis of NOTCH ligands by epsin (58), a protein also over-modified in CLL. In addition, IKAROS (that shows a reduction in its K-GG remnant modification in B-CLL cells compared to control CD19+ peripheral cells) has been shown to antagonize NOTCH signaling (59, 60). These observations may be quite relevant, contributing to the nonmutational NOTCH1 activation found in many CLL patients (61). Further studies are necessary to discern how those modifications contribute to the activation of the NOTCH pathway in CLL.

WNT signaling is nowadays considered as a network of interacting pathways that affect many cellular processes like micro-environment interactions, cytoskeleton rearrangements, cell cycle regulation, proliferation and apoptosis among others. B-CLL cell survival is greatly dependent on stroma and cell to cell interactions, suggesting a key role of WNT signaling in its pathogenesis (62). Indeed, early studies found high expression of genes encoding ligands able to activate the Wnt/ß-catenin pathway (63), although this could not be related to an aggressive form of the disease (64). However, the Wnt/PCP signaling branch, that do not require ß-catenin stabilization, show several of its members up-regulated in B-CLL cells (65). The Wnt/PCP pathway controls the chemotactic response and cell homing in CLL (66) and represents a very promising therapeutic target (62). Indirect data support a role of NEDDylation in the regulation of Wnt signaling, since knockdown of the catalytic subunit of COP9 signalosome, CSN5, leads to decreased ß-catenin levels and to an up-regulation of DKK1 in colorectal carcinoma cell lines (67). Moreover, the NAE inhibitor MLN4924 induces a reduction in DKK1 expression. However, our screening did not identify alterations in the UBL-PTM of any member of this pathway.

Mutations or alterations in the function of p53 are virtually behind all types of tumors. In CLL, deletion of 17p (del17p) or mutation of p53 are the main markers of bad prognosis. Post-translational modifications of p53 play a key role in its function as tumor suppressor and many of them have been described (68). It was somehow surprising to find that only the modification of lysine 120 of p53 was altered in CLL. Lysine 120 is located within de DNA binding domain of p53, and its acetylation has been related to the stabilization of p53 and the increase of its affinity to the promoters of proapoptotic genes (69, 70). Acetylation of lysine 120 has also been related to the induction of apoptosis by p53 through transcriptional-independent mechanisms (71). Either NEDDylation or ubiquitination of p53 impaired its transactivation capacity in luciferase experiments, in concordance with previous reports (72). However, while ubiquitination mediated p53 destabilization, NEDDylation did not. Moreover, we could demonstrate for the first time that NEDDylation of lysine 120 of p53 greatly abolished the acetylation in this residue, along with a loss in the transactivation capacity of p53 and an impairment of its interaction with Bcl-2 (31). These alterations in p53 function support a role of lysine 120 over-NEDDylation in the etiology and prognosis of CLL patients.

## Summary and Future Directions

The recently published UBL-PTM profile of chronic lymphocytic leukemia has placed these transversal processes in a central position between the different intrinsic, genetic and environmental factors that explain the CLL phenotype. Among them, NEDDylation is recently receiving special attention, due to the promising results obtained with its inhibitor, MLN4924, in preclinical assays. MLN4924 blocks the micro environmental activation of NF-kB and induces cell death or senescence in B-CLL cells. However, the UBL-PTM profile of these cells has extended the putative role of NEDDylation over the regulation of many other different pathways, key in the physiopathology of CLL and target of the new therapies used to fight it. However, the limitations of the tools available to date to specifically profile NEDDylation in different pathologies makes mandatory a detailed study of the nature of each of the modifications found as altered. These studies will help us understand the role of these kind of post-translational modifications in the physiopathology of CLL and will surely uncover new therapeutic targets.

## Author Contributions

VA and JC contributed equally to the writing of the introduction to the molecular aspects. JD-G wrote the introduction to the clinical aspects of the disease. LY and CP defined the conception and design of the manuscript, revised and wrote parts of it. All authors contributed to the article and approved the submitted version.

## Funding

This work has been funded by: Project PI17/01688 from Instituto de Salud Carlos III co-financed by European Development Regional Fund (FEDER); Project CI20/30 from Asociación Luchamos por la Vida.

## Conflict of Interest

The authors declare that the research was conducted in the absence of any commercial or financial relationships that could be construed as a potential conflict of interest.

## Publisher’s Note

All claims expressed in this article are solely those of the authors and do not necessarily represent those of their affiliated organizations, or those of the publisher, the editors and the reviewers. Any product that may be evaluated in this article, or claim that may be made by its manufacturer, is not guaranteed or endorsed by the publisher.
